# Sublingual Priming with a HIV gp41-Based Subunit Vaccine Elicits Mucosal Antibodies and Persistent B Memory Responses in Non-Human Primates

**DOI:** 10.3389/fimmu.2017.00063

**Published:** 2017-02-01

**Authors:** Selma Bekri, Pierre Bourdely, Carmelo Luci, Nathalie Dereuddre-Bosquet, Bin Su, Frédéric Martinon, Véronique M. Braud, Irene Luque, Pedro L. Mateo, Sara Crespillo, Francisco Conejero-Lara, Christiane Moog, Roger Le Grand, Fabienne Anjuère

**Affiliations:** ^1^Université Côte d’Azur, Nice, France; ^2^CNRS UMR7275, IPMC, Valbonne, France; ^3^INSERM, Paris, France; ^4^CEA, Université Paris Sud, INSERM U1184 “Immunology of Viral Infections and Autoimmune Diseases”, Fontenay-aux-Roses, France; ^5^INSERM, Unit 1109 INSERM/UNISTRA, Fédération de Médecine Translationnelle de Strasbourg, Strasbourg, France; ^6^Beijing Key Laboratory for HIV/AIDS Research, Center for Infectious Diseases, Beijing You’an Hospital, Capital Medical University, Beijing, China; ^7^Departamento de Química Física e Instituto de Biotecnología, Universidad de Granada, Granada, Spain

**Keywords:** sublingual immunization, mucosal antibodies, B memory response, neutralization, HIV, trimeric gp41, cholera toxin B subunit

## Abstract

Persistent B cell responses in mucosal tissues are crucial to control infection against sexually transmitted pathogens like human immunodeficiency virus 1 (HIV-1). The genital tract is a major site of infection by HIV. Sublingual (SL) immunization in mice was previously shown to generate HIV-specific B cell immunity that disseminates to the genital tract. We report here the immunogenicity in female cynomolgus macaques of a SL vaccine based on a modified gp41 polypeptide coupled to the cholera toxin B subunit designed to expose hidden epitopes and to improve mucosal retention. Combined SL/intramuscular (IM) immunization with such mucoadhesive gp41-based vaccine elicited mucosal HIV-specific IgG and IgA antibodies more efficiently than IM immunization alone. This strategy increased the number and duration of gp41-specific IgA secreting cells. Importantly, combined immunization improved the generation of functional antibodies 3 months after vaccination as detected in HIV-neutralizing assays. Therefore, SL immunization represents a promising vaccine strategy to block HIV-1 transmission.

## Introduction

The genital tract represents a major site of human immunodeficiency virus (HIV)-1 transmission (1, 2). Vaccination protocols aiming at developing systemic IgG antibody or cytotoxic T cell responses were not effective or only partly protective against HIV-1 heterosexual infection, respectively (3, 4). The reasons why these vaccines failed to be protective still remain to be fully established. One can expect that mucosal antibody responses could play a role in protection. Indeed, HIV-specific secretory IgA and IgG were detected in the cervico-vaginal secretions of HIV-exposed uninfected individuals. Such antibodies were described to exert HIV-neutralizing activity or to block HIV transcytosis through an artificial epithelium (5–7). The HIV-1 glycoprotein gp41 undergoes conformational changes leading to membrane fusion and viral entry. It is one target of neutralizing or blocking antibodies. Therefore, the development of vaccines that induce mucosal B cell immunity against selected epitopes of gp41 should protect against HIV-1 infection.

Mucosal immunization, unlike parenteral immunization, favors the generation of B and T cell responses both at the site of mucosal immunization and in distant mucosae (8). Vaginal immunization was shown to induce IgA and IgG antibody-secreting cells in the vagina as well as cervico-vaginal and serum antibodies (9–11). Nasal immunization gave rise to robust IgA and IgG antibody responses in macaque and human genital tracts (10, 12). Nevertheless, there is a risk of antigen passage through the brain barrier after nasal vaccination as experienced with a toxin-based non-living influenza vaccine that can limit the development of nasal subunit vaccines (13). More recently, the sublingual (SL) route for delivering vaccines was shown to generate broadly disseminated mucosal and systemic immune responses (14, 15). Notably, SL immunization in mice was as potent as vaginal immunization for the induction of IgA and IgG antibody and cytotoxic T cell responses in the female genital tract, whereas parenteral immunization did not. It provided protection against genital challenge with HPV pseudovirions (11, 16). Moreover, recent prime-boost vaccine strategies combining mucosal (either nasal or SL) and systemic immunizations were beneficial to generate protection in macaques against a challenge by SHIV (17) or in mice against *Chlamydia* genital shedding (18).

Mucosal immunization with non-replicative antigens requires the use of adjuvants and delivery systems in order to break mucosal tolerance and to facilitate the uptake of immunogens (19). The cholera toxin B subunit (CTB) binds to GM1 gangliosides expressed by all nucleated cells and constitutes a good antigen-delivery vector for covalently linked proteins and polysaccharides (20). Intravaginal or SL application of an antigen covalently coupled to CTB in mice was shown to generate vaginal B and T cell responses (16, 21). The potential of SL immunization in macaques and in humans remains to be established. In the current study, we investigated the immunogenicity in female cynomolgus macaques of a SL prime/intramuscular (IM) boost HIV gp41-based vaccine designed to expose hidden epitopes.

## Materials and Methods

### Vaccine

The trimeric HIV-1 gp41-derived polypeptide (mgp41) was produced by PX’therapeutics (Grenoble, France) and characterized by biophysical analyses as described in Supplementary Material. Several mgp41 amine residues were substituted with sulfhydryl groups using Traut’s reagent (Piercenet, Rockfort, IL, USA) and then coupled to CTB (Crucell AB, Stockholm, Sweden) using the sulfosuccinimidyl 4-[*p*-maleimidophenyl]butyrate crosslinker (Piercenet). The CTB-mgp41 conjugate was purified by ultracentrifugation on AMICON filters and its purity checked by migration on SDS-PAGE gel. Its biological activity and the CTB:mgp41 ratio were measured by solid phase ELISA using GM1 gangliosides as capture system and antibodies to CTB or mgp41 for detection. Cholera toxin (CT) from List Biologicals laboratories (USA) was used as adjuvant for SL immunizations and Aluminum hydroxide (Sanofi-Pasteur, Lyon, France) as adjuvant for IL immunizations.

### Study Design

Eighteen adult female cynomolgus macaques (*Macaca fascicularis*) from Mauritius were housed within CEA animal facilities (Fontenay-aux-Roses, France) according to French national regulations and under the supervision of national veterinary inspectors (CEA Permit Number A 92-032-02). The CEA complies with the Standards for Human Care and Use of Laboratory Animals of the Office for Laboratory Animal Welfare (OLAW, USA) under OLAW Assurance number #A5826-01. This study was approved and accredited under statement number A10-053 by the ethics committee “Comité d’Ethique en Expérimentation Animale du CEA” registered under number 44 by the French Ministry of Research. Animals weighing between 3 and 6 kg, seronegative for SIV, STLV, filovirus, HBV, herpes B, and measles and genotyped for Class I and Class II MHC alleles were used. They were equally distributed between groups based on their genotype (*n* = 6 per group). Animals were sedated with ketamine chlorhydrate (Rhone-Merieux, Lyon, France) for 1 h. Group 1 received five SL immunizations with CTB-mgp41 (100 μg/dose of mgp41 antigen at W0, 4, and 12 and 50 μg/dose at W8 and 20) with CT (10 μg/dose). Group 2 received three SL immunizations with CTB-mgp41 and CT (SL priming) at W0, 4, and 8 similarly to group 1 followed by IM boosts with 100 µg of mgp41 in Alum (500 μg/dose) at W12 and 20. Group 3 received a SL priming with CT alone (10 µg) at W0, 4, and 8 followed by IM boosts with 100 µg of mgp41 in Alum (500 μg/dose) at W12, 20, and 28. The SL vaccine was applied in a 500 µL volume of PBS under the tongue of sedated animals with their head bending forward to avoid leakage of excess of vaccine and was then rinsed with PBS 15 min later in order to avoid swallowing. For intramuscular immunizations, a 500 µL volume was injected in the right thigh for W12, the left thigh for W20, and the right thigh for W28.

### Fluid Harvesting

Blood, vaginal, and rectal washes were collected for antibody analysis by ELISA (W0, W6, W10, W14, W22, W24, and W28). Vaginal and rectal secretions were collected using Weck-cel™ sponges pre-moistened with 100 µL of PBS in presence of protease inhibitors as described previously (22).

### Measurement of Specific Antibodies

Fluids were assayed for mgp41-specific IgA and IgG antibodies by a two-step amplified ELISA using biotin-conjugated goat anti-human IgG or IgA antibodies (Southern Biotech, Birmingham, AL, USA) and HRP-conjugated avidin (Sigma-Aldrich, St. Quentin Fallavier, France). Solid-phase bound HRP activity was monitored spectrophotometrically after addition of enzyme substrate. The endpoint titer was the reciprocal of the sample dilution giving an absorbance at least equal to threefold that of background [corresponding pre-immune sample at the lowest dilution (1/50 for sera and 1/3 for mucosal samples)].

### Cell Isolation

Peripheral blood mononuclear cells (PBMCs) were isolated from blood 5 days after the priming (W9) and the boost (W21 for group 1 and 2 or W29 for group 3), 4 weeks after the last immunization (W24, groups 1 and 2 or W32, group 3), and 3 months after the last immunization (W32, groups 1 and 2 or W40, group 3) and assessed for antibody-secreting cells (ASCs) by B cell ELISPOT assay. Macaques were sacrificed 3 months after the last immunization (W33, groups 1 and 2 and W41, group 3) and rectal and vaginal mononuclear cells (MNCs) were prepared by enzymatic digestion of tissues with collagenase II (0.3 mg/mL) in the presence of trypsin inhibitor (0.1 mg/mL) (Sigma-Aldrich) and DNase I (0.1 mg/mL) (Roche) in RPMI medium supplemented with 3% FBS, 25 mM Hepes, antibiotics and Fungizon^®^ according to supplier’s recommendations (Invitrogen, Saint Aubin, France). Bone marrow (BM) MNC from iliac crest or from humerus were isolated using Ficoll density-gradient centrifugation (MSL2000, Eurobio, Les Ulis, France). PBMC and tissue-derived MNC collected 1 or 3 months after immunization were stimulated *in vitro* for 6 days with Pokeweed Mitogen (PWM, 1 µg/mL) and *Staphylococcus aureus* Cowan strain (SAC, 0.1 µg/mL) (Sigma-Aldrich) before ASC enumeration.

### B Cell Elispot Assay

Peripheral blood mononuclear cell and MNC from vagina and rectum were assayed for mgp41-specific ASC using an amplified B ELISPOT assay as described (23). Briefly, graded numbers of cells (between 1 × 10^5^ and 8 × 10^5^) were incubated for 16 h at 37°C in nitrocellulose-bottom 96-well plates coated with mgp41 (5 µg/mL) for detection of mgp41-specific ASC or with mouse anti-human kappa and lambda antibodies (5 µg/mL) (Southern Biotech, Birmingham, AL, USA) for detection of total ASC. The antibodies produced were detected by stepwise incubations with biotin-conjugated goat anti-human IgG or IgA (KPL, Gaithersburg, MD, USA), avidin-conjugated peroxidase (Sigma-Aldrich), and AEC-H_2_O_2_ chromogenic peroxidase substrate (Sigma-Aldrich). Spots were enumerated using CTL immunospot analyzer (Bonn, Germany).

### Flow Cytometry

Mononuclear cells were incubated with fluorescent monoclonal antibodies against surface markers in order to identify B cell subsets. Antibodies from BD Biosciences used: anti-CD45 (clone D058-1283), anti-CD3 (clone SP34-2), anti-HLA-DR (clone L243), anti-CD20 (clone 2H7), as well as anti-CD27 (clone M-T271, Miltenyi) and anti-IgD (rabbit polyclonal, AbDserotec). Cells were analyzed on BD LSRII FACS analyzer and Diva Software (BD Biosciences).

### Immunostainings

Cryosections of SL mucosa were fixed with acetone before hematoxylin/eosin staining according to supplier’s instructions (Sigma-Aldrich) and analyzed using a fluorescence microscope DMD108 (Leica, Nanterre, France). Cryosections were stained with purified antibodies against HLA-DR-DP-DQ (clone CR3/473) and CD1a (clone O10) (Dako, Les Ulis, France) or corresponding isotype controls, revealed with donkey anti-mouse AlexaFluor 594 (Invitrogen), and analyzed with Zeiss LSM780 confocal microscope (Marly Le Roi, France).

### Virus Neutralization Assay

Neutralization assays were performed as described (24) using two standard reference strains as Env-pseudotyped viruses (SF162.LS, tier 1 and QH0692.42, tier 2) to infect TZM-bl cells. The 50% inhibitory dose was defined as the sample dilution that caused a 50% reduction in relative luminescence units (RLUs) (25).

### Statistical Analyses

Statistical analyses were carried out with Graphpad prism version 6.0 (La Jolla, CA, USA). Pairwise multiple comparisons of experimental groups were performed using the non-parametric Mann–Whitney *U*-test.

## Results

### Vaccine Characterization

**Figure 1:**
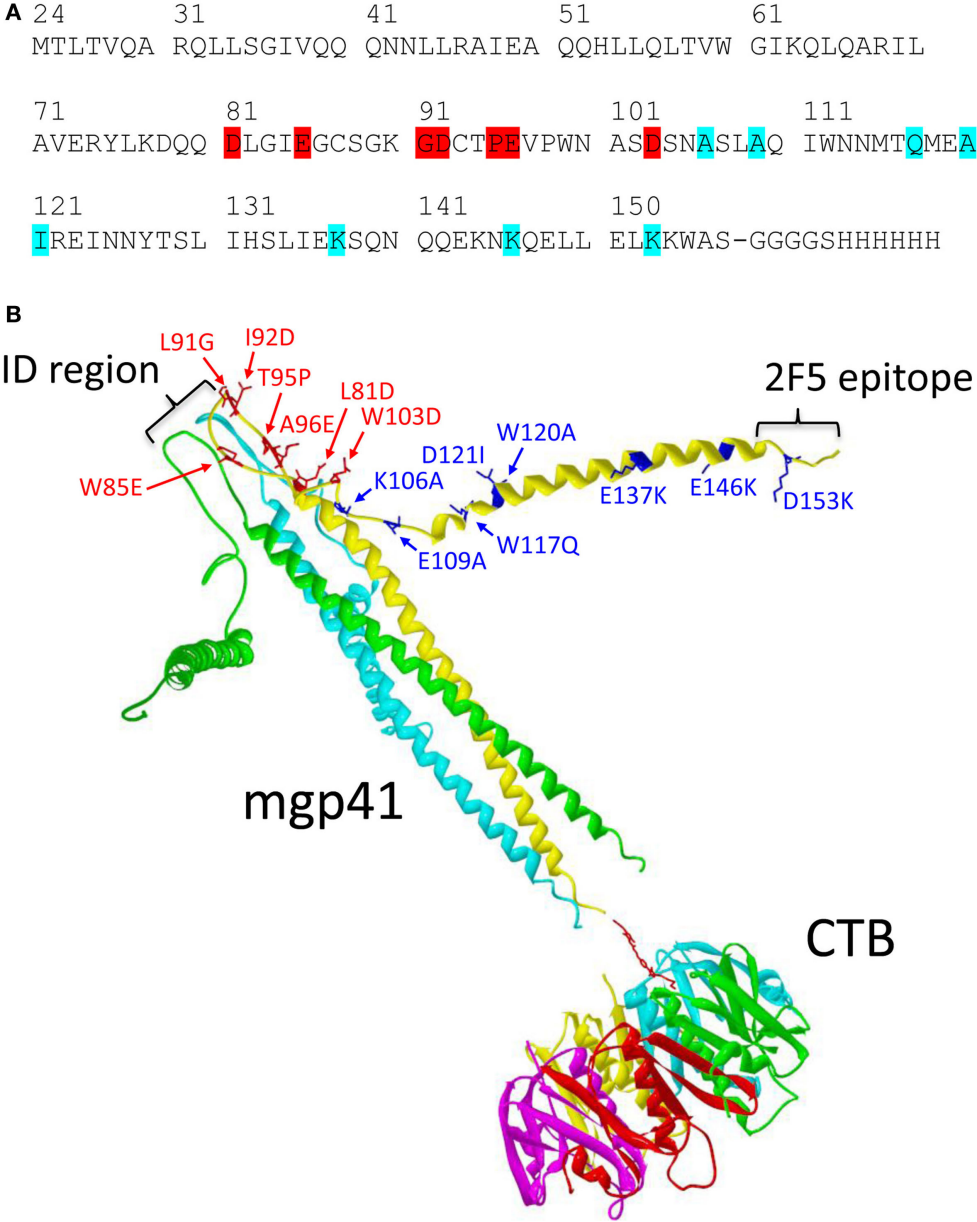
**Mgp41-cholera toxin B subunit (CTB) vaccine construct used for sublingual immunizations**. **(A)** Linear sequence of the human immunodeficiency virus (HIV)-1 gp41 fragment including punctual mutations designed to increase antigen solubility under physiological conditions (highlighted in red) and to potentiate exposure of hidden conserved epitopes in gp41 (highlighted in cyan). **(B)** Schematic ribbon representation of one mutated gp41 polypeptide fragment in a trimeric form (mgp41) chemically linked to a pentamer of CTB used as mucosal antigen delivery vector as detailed in Section “Materials and Methods.” The picture was made using Swiss PDB viewer (30) using the model structures of the HIV gp41 ectodomain (PDB code 1IF3) (31) and the cholera toxin B-pentamer (PDB code 1CHB) (32). The spatial orientation of the C-terminal helical region helices of mgp41 was manually altered to indicate disruption of the six-helix bundle structure. Mutated residues in mgp41 are highlighted as sticks in one of the monomers. The locations of the immunodominant region and the 2F5 epitope region are also indicated on one of the gp41 monomers. Each polypeptide monomer is represented in different colors for clarity. The divalent cross-linker is represented with red sticks. The resulting subunit vaccine [called cholera toxin B subunit (CTB)-mgp41] contains one trimeric gp41 per CTB pentamer.

A vaccine containing a gp41 polypeptide conjugated to the CTB mucoadhesive vector was designed to improve its SL delivery (Figure 1). We used a gp41 polypeptide including the gp41 amino acids M24 to S157 from the gp41 sequence of the BRU/LAI isolate in which several mutations were added (Figure 1A) (26). Mutations of seven residues were introduced in the gp41 loop region to increase its net charge and reduce its hydrophobicity, thus reducing the propensity to aggregation of the gp41 ectodomain (27). In addition, eight additional mutations were made at the C-terminal helical region (CHR) to open the six-helix bundle structure and to expose hidden epitopes at the conserved N-terminal helical region (NHR). Dynamic light scattering analyses revealed that the mutated gp41 (called hereafter mgp41) was trimeric in solution. Circular dichroism measurements indicated that around 66% of the mgp41 structure was in alpha-helices and that its thermal stability was strongly reduced compared to the native gp41 ectodomain (Figure S1 in Supplementary Material) (28), suggesting a destabilization of the six-helix bundle. Accessibility of NHR epitopes was confirmed by the significant binding of an exogenous CHR peptide to mgp41 (gp41 sequence 110-141) (29) (Figure S1 in Supplementary Material). The mgp41 polypeptide was chemically conjugated to CTB using a strategy allowing the coupling of one mgp41 per CTB pentamer as illustrated in Figure 1B.

### Vaccine Strategy in Macaques

**Figure 2:**
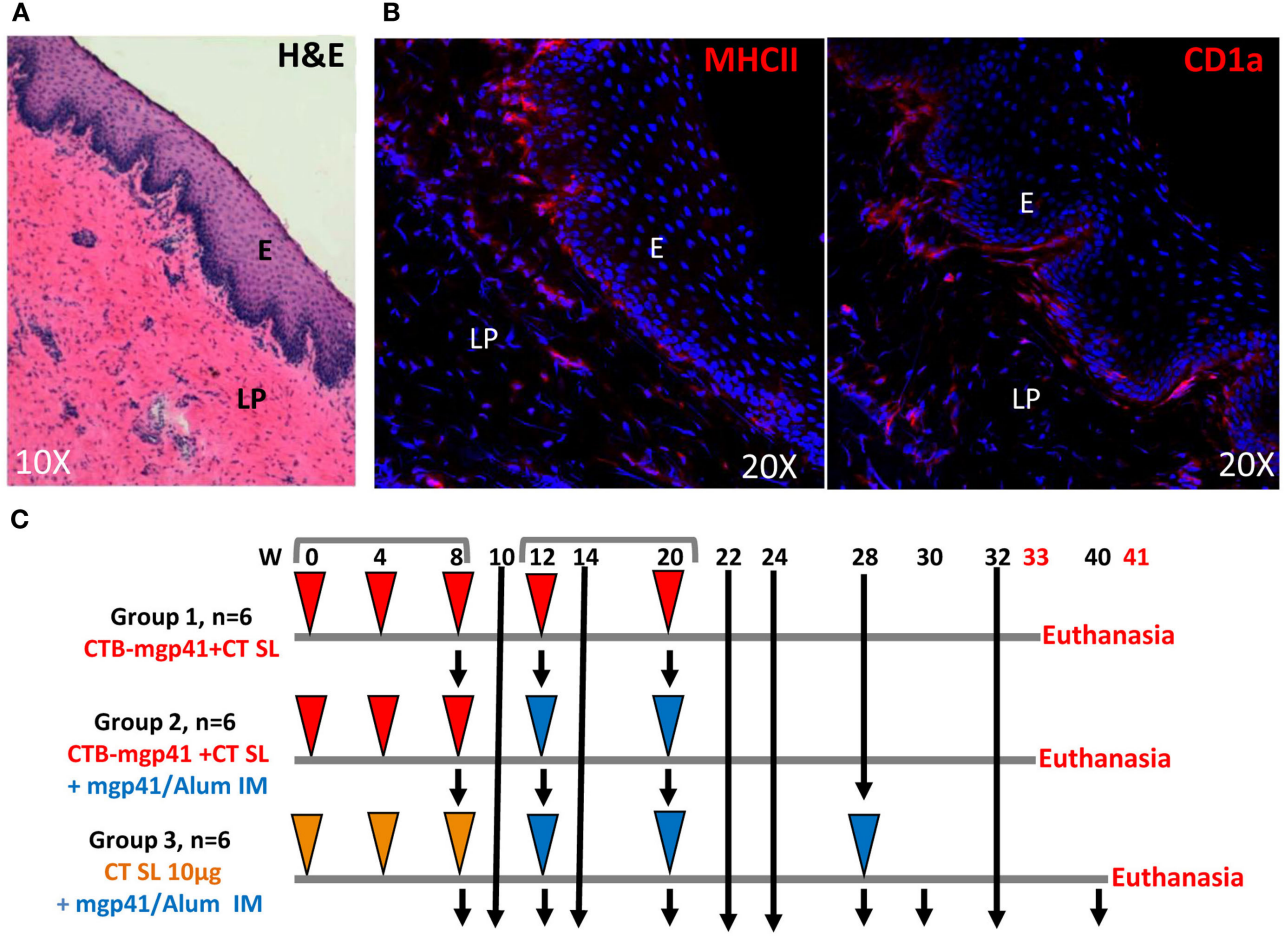
**Vaccination strategy**. **(A,B)** Cryosections analyses of the macaque sublingual (SL) tissue. **(A)** Haematoxylin eosin staining of the SL mucosa from a control cynomolgus macaque. **(B)** SL distribution of MHCII^+^ cells (MHCII^+^, left panel) and CD1a^+^ cells (right panel) by immunofluorescence. Nuclei are stained with Dapi (blue color). E, epithelium; LP, lamina propria. **(C)** Female cynomolgus macaques were immunized at weeks (W) 0, 4, 8, 12, and 20 either with cholera toxin B subunit (CTB)-mgp41 together with cholera toxin (CT) (red arrowheads) or CT alone (orange arrowheads) by SL route or with mgp41 in alum by intramuscular (IM) route (blue arrowheads). Macaques from group 1 were immunized sublingually with CTB-mgp41 together with CT at the doses indicated in Section “Materials and Methods.” Macaques from group 2 received a SL priming immunization at W0, 4, and 8 similar to group 1 followed by an IM boost immunization with 100 µg of mgp41 in Alum at W12 and 20. Macaques from group 3 received a priming SL immunization with CT alone at W0, 4, and 8 followed by three IM boost immunizations (W12, 20, and 28). Black arrows correspond to sample collections. Vaginal, rectal secretions, and sera were collected throughout the protocol for antibody analysis by ELISA. Peripheral blood mononuclear cells (PBMCs) were prepared for B ELISPOT analysis from blood of pre-immune macaques, blood of immunized macaques collected 5 days after priming (W9), 5 days after boost (W21), and 3 months after boost (W32 or W40). Macaques were sacrificed 1 week later (W33 or W41) and PBMC as well as bone marrow cells were prepared for memory B cells analysis by flow cytometry.

The immunogenicity of the SL CTB-mgp41 vaccine was evaluated in female cynomolgus macaques. Immunohistochemistry analysis confirmed that the macaque SL mucosa was an immunocompetent tissue with a pluristratified epithelium underlined by a thin submucosa containing MHCII^+^ antigen-presenting cells, some of them being CD1a^+^ dendritic cells (DCs) (Figures 2A,B). We compared the efficacy of the SL route of vaccination to the IM immunization and to a combined strategy including SL priming followed by IM boost (SL/IM) to generate serum and mucosal IgG and IgA antibodies against mgp41. As illustrated in Figure 2C, six macaques (group 1, SL) were immunized sublingually with CTB-mgp41 together with CT used as mucosal adjuvant. Six macaques (group 2, SL/IM) received a SL priming similarly to group 1 followed by IM boosts. In order to evaluate the impact of the SL priming in the combined SL/IM vaccination, the six macaques from group 3 (IM) were primed with CT alone by SL immunization and received IM boosts with mgp41 in Alum. Furthermore, macaques from group 3 received a third IM immunization in order to compare SL and SL/IM strategies to optimized conditions for IM vaccination.

### SL Priming Immunization Improves Antigen-Specific Antibody Responses

**Figure 3:**
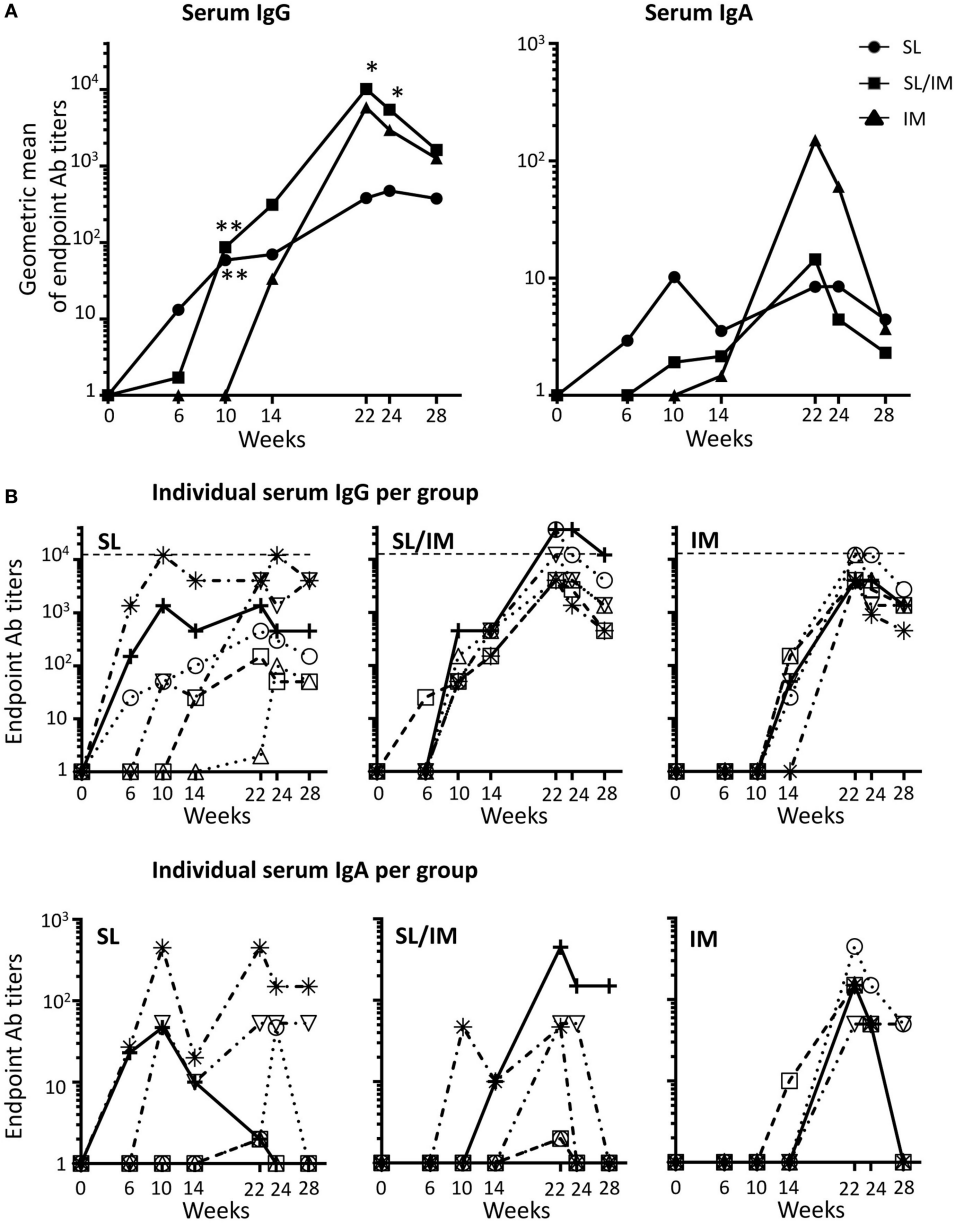
**SL/IM combined immunization increases mgp41-specific IgG antibody responses in serum**. Macaques were immunized according to the study design detailed in Figure 2C. SL referred to group 1, SL/IM to group 2, and IM to group 3. Mgp41-specific IgG and IgA antibodies were detected in macaque sera at indicated time points (from week 0 to week 28) by ELISA. **(A)** Data represent the geometric means of endpoint titers measured at indicated time points for each immunization group (*n* = 6 per group). * and ** in graphs denote statistical differences between SL group or SL/IM group and IM group using the non-parametric Mann–Whitney *U*-test, *p < 0.05 and **p < 0.005. **(B)** Graphs correspond to titers from individual sera (*n* = 6) at the indicated time points for each immunization group (group 1, left graph; group 2, middle graph; group 3, right graph).

**Figure 4:**
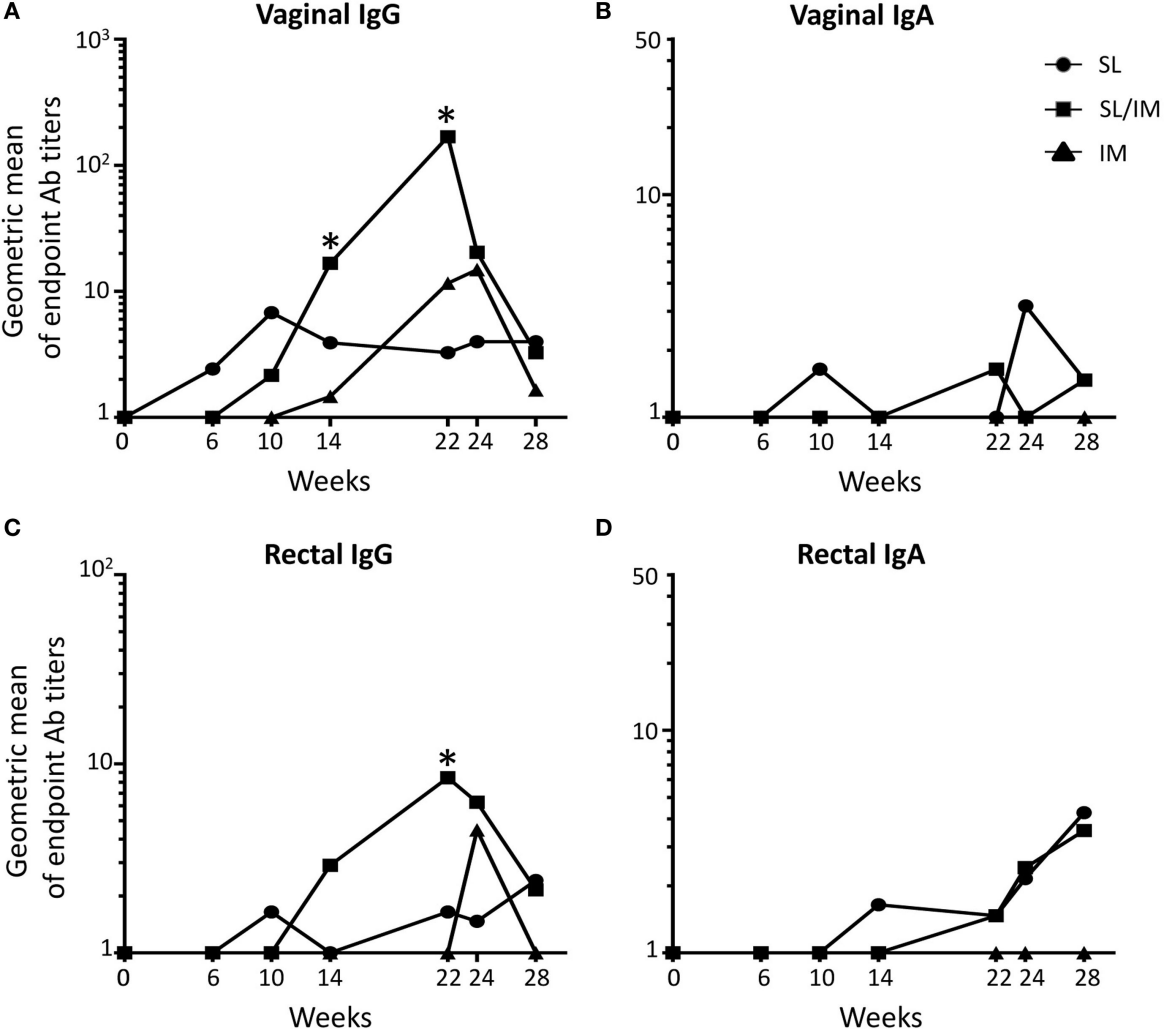
**Sublingual (SL) priming increases mgp41-specific antibody responses in vaginal and rectal fluids**. Macaques were immunized according to the study design detailed in Figure 2C. SL referred to group 1, SL/IM to group 2, and IM to group 3. IgG **(A,C)** and IgA **(B,D)** antibodies specific for mgp41 were detected in vaginal **(A,B)** and rectal fluids **(C,D)** obtained from individual macaques at the indicated time points. **(A–D)** Graphs represent the geometric means of endpoint titers measured at indicated time points for each immunization group. * in graphs denotes statistical differences between SL/IM group and IM group with *p* < 0.05 using the non-parametric Mann–Whitney *U*-test.

To compare the potential of SL immunization to classical IM vaccination, we quantified the mgp41-specific antibodies of IgG and IgA isotypes generated throughout the protocol. Antigen-specific antibodies were measured in sera (Figure 3) and in vaginal and rectal washes (Figure 4) at the indicated time points. The macaques that received five SL immunizations had notable serum mgp41-specific IgG titers up to 2 months after the last immunization (Figure 3A, left panel; Figure 3B, upper panel). Serum mgp41-specific IgG titers reached a significant value after the third SL immunization but the titers did not increase with subsequent SL immunizations (Figure 3A, left panel), suggesting that repeated immunizations with 1-month intervals reduced antigen uptake. In contrast, boost IM immunizations in the SL/IM strategy increased serum mgp41-specific IgG titers by around 10-fold compared to SL immunization (Figure 3A). Interestingly, SL/IM immunization gave significantly higher mgp41-specific IgG antibody levels than the IM strategy 2 weeks and 1 month after the fifth immunization, indicating that SL priming potentiated antibody responses (Figure 3A). No statistical differences in serum mgp41-specific IgA titers were detected between immunization groups (Figure 3).

Vaginal and rectal antibody levels induced by the different vaccine strategies were low (Figure 4). All the macaques had mgp41-specific IgG in vaginal washes (Figure 4A), suggesting that these antibodies were produced systemically. Accordingly, rare mgp41-specific IgG ASCs were detected in the genital tract of macaques (data not shown). Interestingly, the SL/IM immunization induced significantly higher levels of gp41-specific IgG antibodies compared to the IM immunization 2 weeks after the fourth and fifth immunizations both in the genital tract and the rectal mucosa (Figures 4A,C). Furthermore, mgp41-specific IgA antibodies were detected in the genital tract and in the rectal mucosa after SL and SL/IM vaccination, but not after IM immunization (Figures 4B,D). Of note, the individuals who developed vaginal responses were the same who developed rectal responses (data not shown). Furthermore, vaginal gp41-specific IgA antibodies detected in SL and SL/IM groups were associated with higher numbers of vagina-associated mgp41-specific IgA ASC in these groups compared to IM immunization (SL: 7 ± 3 gp41-specific IgA ASC; SL/IM: 8 ± 2 gp41-specific IgA ASC; IM: 4 ± 2 gp41-specific IgA ASC).

### Combined SL/IM Immunization Increases the Frequency and Persistence of B Cell Responses

**Figure 5:**
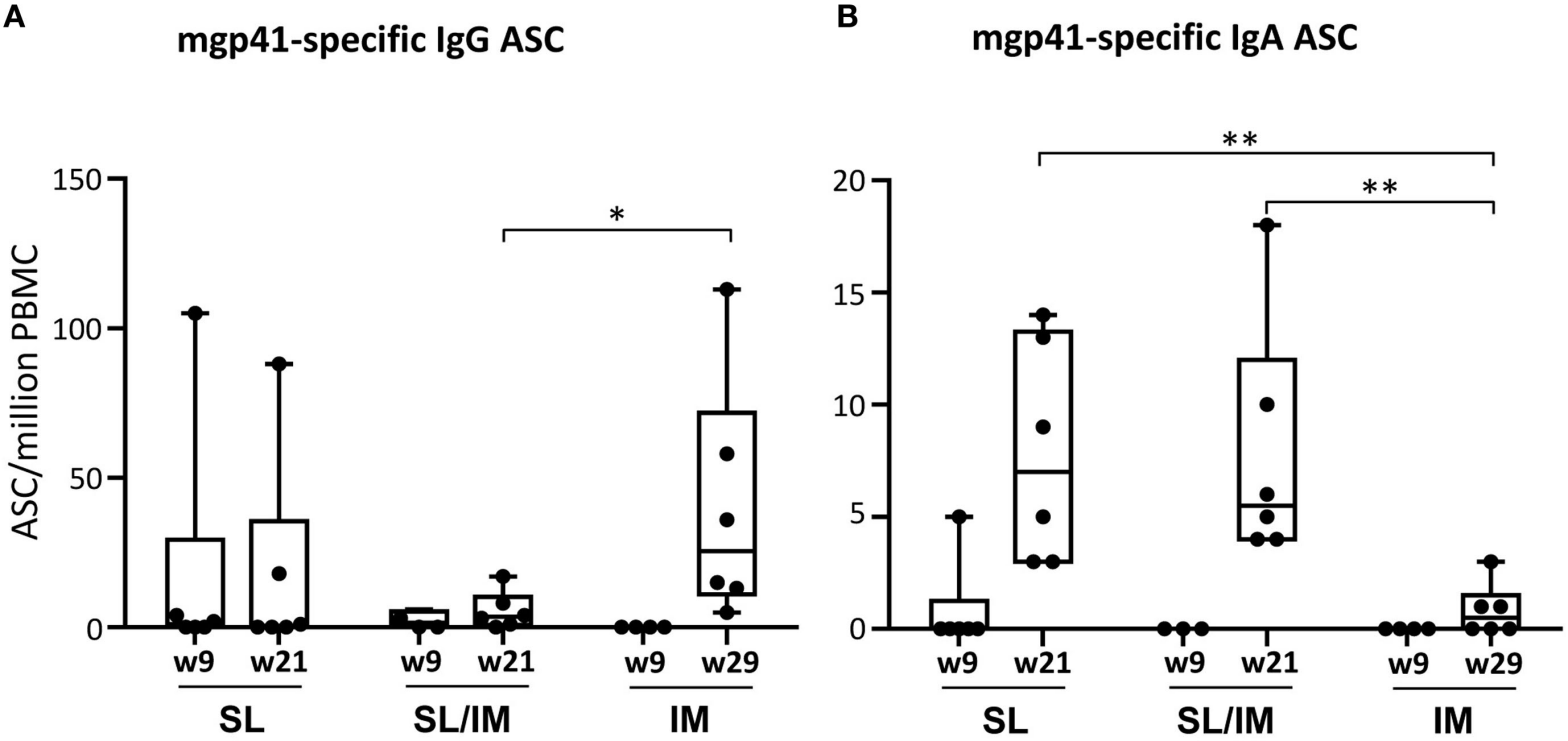
**Combined SL/IM immunization increases the numbers of circulating mgp41-specific IgA antibody-secreting cells (ASCs)**. Macaques were immunized according to the study design detailed in Figure 2C. SL referred to group 1, SL/IM to group 2, and IM to group 3. Peripheral blood mononuclear cells (PBMCs) were prepared from blood of individual macaques collected 5 days after priming immunizations (W9) and boost immunizations (W21 or W29 for group 3). Frequency of circulating mgp41-specific ASC of IgG and IgA isotypes was obtained by B ELISPOT assay. Results are represented in boxes and whiskers as individual numbers of mgp41-specific ASC of IgG **(A)** and IgA **(B)** isotypes and as the median value of gp41-specific spot numbers per million PBMC at the indicated time points for each immunization group (*n* = 6). * and ** in graphs denote statistical differences with *p* < 0.05 and *p* < 0.01, respectively, using the non-parametric Mann–Whitney *U*-test.

To further analyze the antibody responses induced after SL and SL/IM immunizations compared to IM immunization, the frequency of circulating mgp41-specific ASC of IgG and IgA isotypes was determined 5 days postimmunization after priming (W9), boost (W21), and third IM immunizations (W29 only for IM group) (Figure 5). Variable numbers of mgp41-specific IgG ASC between macaques of a same group were detected after the priming with no significant differences between groups. The macaques from IM group had significantly higher numbers of circulating IgG ASC after the third IM immunization (week 29) compared to the other groups (Figure 5A), which can be explained by the increased bioavailability of the antigen after IM immunization. In contrast, both SL and SL/IM immunizations induced higher numbers of mgp41-specific IgA ASC after the last immunization compared to the IM group (Figure 5B), confirming in macaques the importance of the route of immunization to induce antibody responses of IgA isotype.

**Figure 6:**
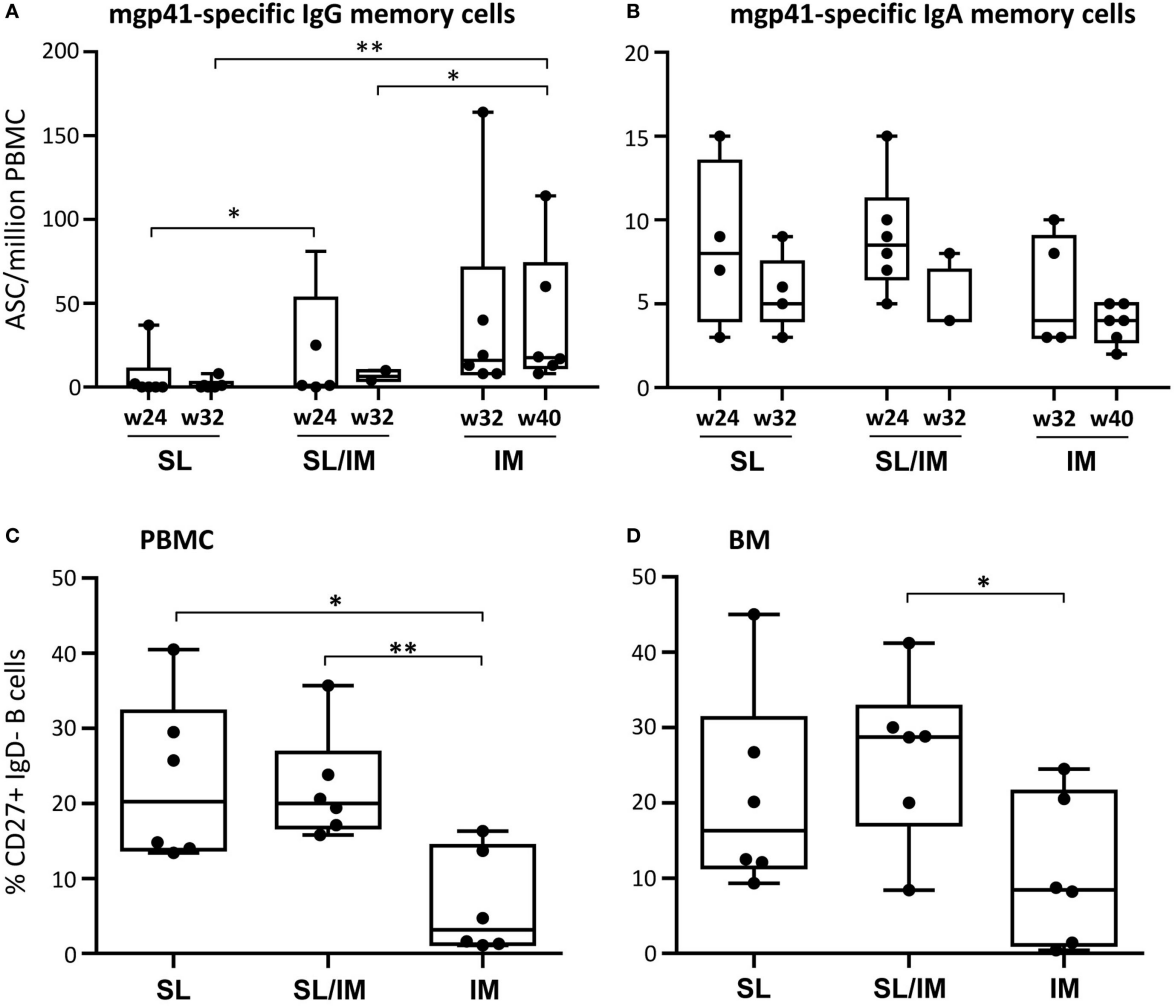
**Sublingual (SL) immunization induces persistent gp41-specific IgA antibody responses and increased memory B cells**. **(A,B)** Peripheral blood mononuclear cells (PBMCs) collected 4 weeks (W24 for SL and SL/IM groups and W32 for IM group) and 3 months after the last immunization (W32 for group 1 and 2 and W40 for group 3) were stimulated *in vitro* as described in Section “Materials and Methods.” The frequency of circulating mgp41-specific antibody-secreting cell of IgG **(A)** and IgA **(B)** isotypes was measured by B ELISPOT assay. Results are expressed as individual values in boxes and whiskers with median values for each immunization group. * and ** in graphs denote statistical differences with *p* < 0.05 and *p* < 0.005, respectively, using the non-parametric Mann–Whitney *U*-test. **(C,D)** The frequency of total CD27^+^IgD^−^ memory B cells among CD20^+^ B cells was assessed by flow cytometry in blood **(C)** and bone marrow cells **(D)** of individual macaques at the end of the protocol according to the gating strategy shown in Figure S2 in Supplementary Material. Results are represented as percentages of CD27^+^IgD^−^ memory B cells among CD20^+^ B cells. * and ** in graphs denote statistical differences with *p* < 0.05 and *p* < 0.005, respectively, using the non-parametric Mann–Whitney *U*-test.

To further evaluate the persistence of the gp41-specific responses generated by the SL mgp41-CTB vaccine, circulating gp41-specific ASC were also quantified 1 month and 3 months after the last immunization after *in vitro* stimulation in order to enumerate antigen-specific memory B cells. As shown in Figure 6A, the macaques of the IM group had significantly higher numbers of circulating IgG ASC 3 months after the third IM immunization compared to other groups (Figure 6A). In contrast, there seemed to be a trend toward higher numbers of mgp41-specific ASC of IgA isotype 1 month after SL/IM immunization compared to IM immunization as indicated by a *p*-Value of 0.08 using the non-parametric Mann–Whitney *U*-test (Figure 6B), consistent with the observation that SL/IM immunization, but not IM immunization generated mucosal gp41-specific IgA antibodies (Figure 4). We also determined the frequency of total CD20^+^CD27^+^ IgD^−^ memory B cells in the blood and BM at the end of the protocol by flow cytometric analysis. SL and combined SL/IM immunizations strongly increased the frequency of memory B cells both in blood and BM compared to IM immunization (Figures 6C,D).

Altogether, these data indicated that the SL immunization increases the frequency and the duration of IgA antibody responses.

### Combined SL/IM Increases the Neutralizing Activity of Antibodies

**Figure 7:**
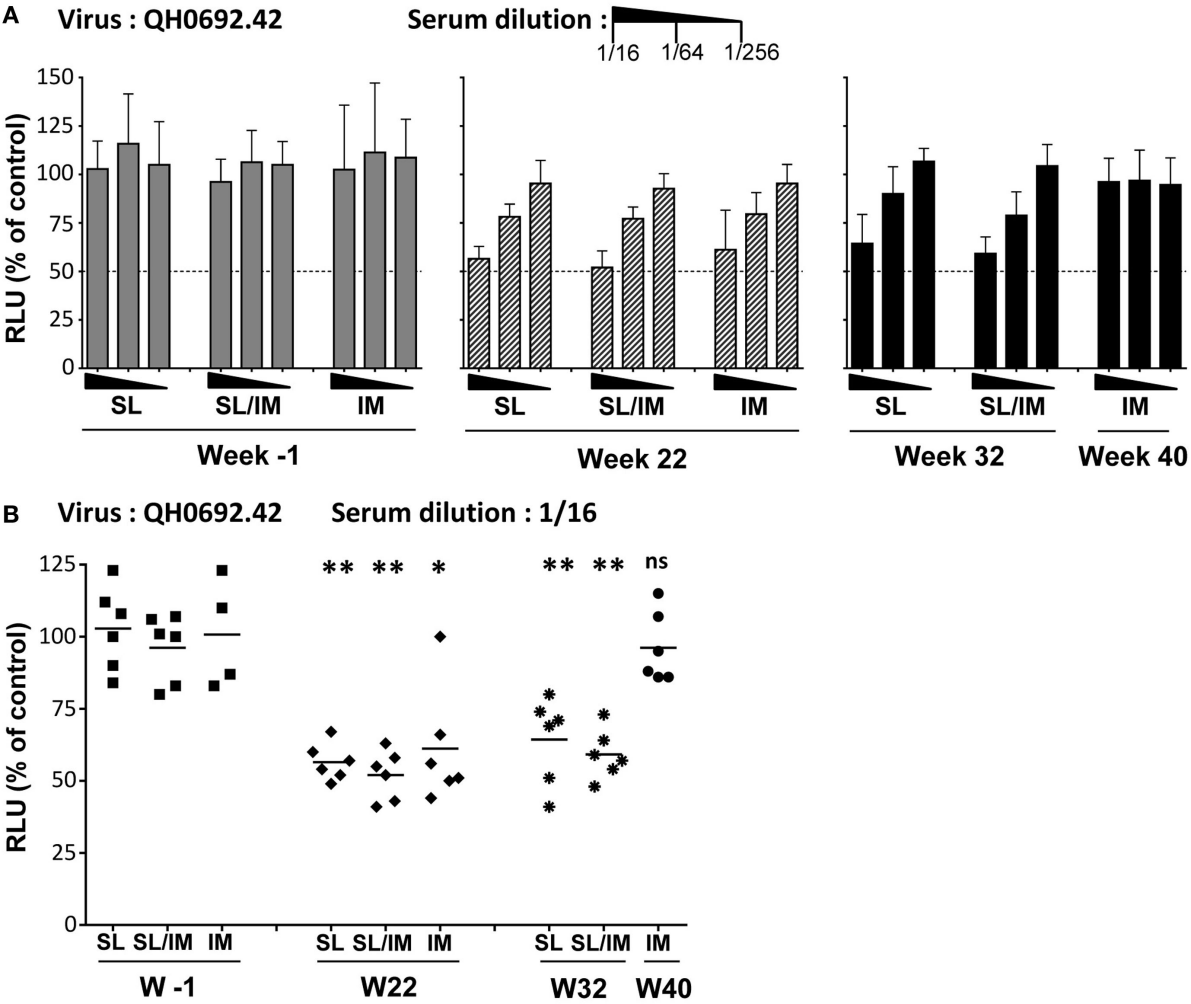
**Neutralizing activity of the sera from immune macaques**. **(A,B)** The human immunodeficiency virus neutralizing activity was performed using pre-immune (W-1) and immune sera 2 weeks after the fifth immunization (W22 for all groups) or 3 months after the last immunization (W32 for SL and SL/IM groups, W40 for IM group). The neutralizing activity of immune sera and control sera was performed using a TZM-bl assay and QHO692.42 clade B virus primary isolate. **(A)** Dose–response curve of neutralizing activity of serial dilutions of individual immune sera collected W22 (hatched black bars), W32 or W40 (black bars) were compared to pre-immune sera from the same macaques (gray bars). Bars indicate mean values of the percentage of reduction in infection compared to the reference control without serum + SD. **(B)** Individual neutralizing activity of sera from individual macaques tested at a dilution of 1/16. The results are represented as individual values and mean values for each group. Neutralizing activities were measured twice in independent experiments for each sample. The hatched line indicates a neutralizing activity with IC50. * and ** denote statistical differences between pre-immune sera (W-1) and immune sera (W22, W32) of a same immunization group using the non-parametric Mann–Whitney *U*-test, *p < 0.05 and **p < 0.005; ns, no statistical difference.

To further evaluate the functional characteristics of the antibodies produced by SL immunization compared to antibodies generated after IM immunization, we determined their neutralizing activity against clade B virus primary isolates. The neutralizing activity of sera was performed using a TZM-bl assay with the QHO692.42 virus primary isolate, a clade B transmitted founder virus classified as Tier 2. Sera collected 2 weeks (W22) and 3 months (W32 for SL and SL/IM groups and W40 for IM group) after the last immunization were tested at several dilutions. Pre-immune sera from each macaque were used as control (Figure 7). Dose–response curves indicated that a modest but antigen-specific neutralizing activity was detected in the three immunization groups at week 22 compared to pre-immune samples (Figure 7A). Interestingly, immune sera from SL and SL/IM immunization groups had persistent neutralizing activity 3 months postimmunization contrary to the IM group. Analysis of the sera of individual macaques at the lowest dilution confirmed that the SL priming with the mgp41-CTB antigen improved the functional characteristics of the antibodies produced (Figure 7B). These data indicated that the SL immunization favors the generation and persistence of functional antibodies.

## Discussion

The SL vaccination is attractive against mucosal infections including heterosexual transmission of HIV as recently explored in mouse models (8). The current report demonstrates in non-human primates that SL priming immunization followed by IM boost with a modified gp41-based vaccine increased antigen-specific serum IgG, mucosal IgG and IgA responses and frequency and duration of antigen-specific ASC of IgA isotype. Interestingly, macaques that received a combined mucosal-systemic immunization exhibited functional antibodies 3 months after immunization whereas macaques immunized only by IM route did not. The present data highlight the efficacy of the SL immunization to generate mucosal antibody responses in non-human primates.

We designed a mutated gp41 polypeptide with increased solubility to improve the synthesis process of the vaccine as well as its accessibility to the immune system. The destabilization of the gp41 six-helix bundle structure with mutations allowed the exposure of hidden gp41 epitopes, known to be accessible during virus fusion (33, 34). Moreover, induction by vaccination of HIV broadly neutralizing antibodies (bnAb), such as 2F5, was shown to be difficult due to their autoreactivity with self-antigens leading to immune tolerance (35). Besides, generation of a 2F5-like neutralizing response was described to be dependent on the membrane environment, which is not the aim of our antigen design (36). Therefore, the point mutation of the immunodominant ELDKWA epitope recognized by the bnAb 2F5 was designed to favor the generation of other functional antibodies.

Not only the vaccine design but also the route of immunization are critical to generate the right immunity at the right site (37, 38). We previously reported that the SL application of antigens with CT in mice induced mucosal antibodies and CTL in the systemic compartment, in the upper aerodigestive tract, in the lung mucosa (39), and in the genital tract (11, 16). The present study extends these observations to female macaques.

The present data further indicate that the antibodies produced are functional and exhibit modest neutralizing activity against the Tier 2 virus QH0692.42, which is difficult to neutralize. Antibodies with HIV-blocking transcytosis activity, FcγR-mediated inhibition capacity or antibody-dependent cellular cytotoxicity (ADCC) were recently described to contribute to the protection against HIV infection (17, 40). Further studies are needed to evaluate whether the antibodies produced by the current SL vaccine display additional HIV-blocking inhibitory activity such as ADCC or decreased infectivity potential by aggregation. These latter inhibitory effects would be particularly valuable to protect against HIV-1 mucosal transmission (41).

Combined IM/intranasal vaccination in rhesus macaques with another gp41-based vaccine was shown to induce mucosal antibodies and protection against SHIV vaginal challenges (17). Moreover, SL priming immunization with a replicating adenovirus followed by an IM boost was shown to induce mucosal IgA antibodies and delayed viremia against rectal challenges with SIV_mac251_ in rhesus macaques (42). The present data showing that SL priming with a non-replicative vaccine improved the generation of mucosal antibodies, especially mucosal IgA, extend these observations. Several studies in highly exposed, persistently HIV-1 seronegative (HEPS) female sex workers have shown the presence of mucosal IgA against gp41 in their genital tract (7, 43). Even detected at low levels, purified IgA antibodies from such HEPS volunteers showed neutralizing activity (44, 45). In our study, the SL and combined immunizations, but not the IM immunization, induced antigen-specific mucosal IgA antibodies and IgA-secreting cells. The protective efficacy of such antibodies against viral challenge in macaques remains to be established. If efficient, such a SL vaccine would avoid the potential danger of nasal immunization, which is the passage of antigen through the blood–brain barrier.

Repeated SL immunizations did not increase antibody titers, but just maintained their levels. This suggests that monthly SL immunizations do not amplify the B cell response but rather induce the maturation and functionality of antigen-specific B cell clones as shown after oral (46) and systemic immunizations (47).

The CTB delivery vector used in the present study favors the mucosal delivery of antigens after SL and intravaginal immunizations (21, 48). The SL administration in healthy female volunteers of the licensed quadrivalent HPV vaccine (Gardasil^®^) without any known mucoadhesive properties failed to induce systemic and mucosal antibodies contrary to the IM route, which may be explained by the lack of appropriate delivery system for SL immunization (49). Nevertheless, in the present study, the heterogeneity in antibody responses between macaques immunized by the SL route could reflect an inconsistent mucosal uptake of the vaccine due to its dilution by the saliva of certain macaques. Semi-solid formulations of SL vaccines could certainly improve antigen absorption and immune responses. Such solid formulations could be based on patches, pastilles, or gels that have been recently developed for SL administration of drugs (50, 51). Future SL vaccines might also target local antigen-presenting cells. In fact, the macaque SL mucosa is an immunocompetent tissue containing DCs. SL DCs were shown to be key antigen-presenting cells in mice with enhanced migratory properties contributing to genital immunity (48). Altogether, this suggests that an optimized formulation and the targeting of SL DCs in macaques and humans could improve sublingually induced mucosal immune responses.

In conclusion, such combined SL/IM vaccine strategy improves the quality of the antibodies generated and suggests that the SL/IM vaccination with appropriate formulations of antigens could help to block heterosexual transmission of HIV by increasing B cell immune responses in mucosal tissues.

## Ethics Statement

Animal studies were performed within CEA animal facilities (Fontenay-aux-Roses, France) accordingly to European guidelines for animal care.

## Author Contributions

SB, ND-B, FC-L, CM, RG, and FA designed the experiments. SB, FC-L, CM, RG, and FA interpreted most of the data presented. SB, PB, CL, BS, FM, VB, IL, PM, and SC contributed to several experiments and/or to their interpretation. SB, FC-L, CM, and FA contributed to the paper writing and FA directed the study.

## Conflict of Interest Statement

The authors declare that the research was conducted in the absence of any commercial or financial relationships that could be construed as a potential conflict of interest.
