# Supplementary material for: Sublingual Priming with a HIV gp41-Based Subunit Vaccine Elicits Mucosal Antibodies and Persistent B Memory Responses in Non-Human Primates
Source: Front Immunol. 2017 Feb 1;8:63. doi: 10.3389/fimmu.2017.00063 (PMC5285372; doi:10.3389/fimmu.2017.00063)
Supplement: Supplementary file 1 [file Data_Sheet_1.DOCX]

**SUPPLEMENTARY FIGURES**

**Supplementary Figure 1**. Biophysical characterization of mgp41. (A) Far-UV CD spectrum of mgp41 indicates α-helical structure. (B) Thermal stability is strongly decreased by mutations destabilizing the 6-helix bundle according to differential scanning calorimetry (DSC) analysis. The melting transition of mgp41 (black) appears broadened and shifted to lower temperature as compared with the melting of a wt gp41 ectodomain (red) and a gp41 ectodomain with only 7 mutations at the loop region (blue), both of which have a highly stable 6-helix bundle structure. Near UV CD spectrum of an equimolar mixture between mgp41 and an exogenous gp41 peptide (gp41 sequence 110-141) [[1](#_ENREF_1)] corresponding to the C-terminal helical region, compared to the isolated mgp41 and a wild type gp41 ectodomain. (C) The negative ellipticity band at 292 nm and the positive band at 265 nm are characteristic of the 6-helix bundle conformation of gp41 [[2](#_ENREF_2)]. These bands are not present in mgp41, whereas addition of the gp41 sequence 110-141 restores partially the bands indicating accessibility of the N-terminal helical region of mgp41.

**Supplementary Figure 2**. Gating strategy corresponding to the flow cytometric analysis of CD27^+^IgD^-^ memory B cells among CD20^+^ B cells in the PBMC of individual macaques at the end of the protocol.

**SUPPLEMENTARY MATERIALS AND METHODS**

**Production and biophysical characterization of mgp41**

The genes encoding the mgp41 vaccine were synthesized by Geneart (Regensburg, Germany) and inserted into a pM1800 plasmid. The sequence contained a C-terminal 6xHis-tag to facilitate purification by Ni-affinity chromatography. Mgp41 was produced by E. coli expression and purified to homogeneity by PX’therapeutics (Grenoble, France). Purity (>95%) was checked by silver-stained SDS-PAGE, mass spectrometry analysis, western-blotting and N-terminal sequencing. For biophysical characterization mgp41 solutions were prepared in 20 mM glycine/HCl buffer, pH 2.5. The oligomerization state of mgp41 was measured by dynamic light scattering (DLS) using a Dynapro MS-X instrument (Wyatt, Santa Barbara, CA). The hydrodynamic radius of 3.9 nm is consistent with a trimeric oligomerization state. Circular dichroism spectra were measured in a Jasco 715 spectropolarimeter (Tokio, Japan). Spectra were recorded using a 1 mm path length cuvette in the far-UV region (260-200 nm) and a 5 mm pathlength cuvette in the near-UV region (350-250 nm). Baseline spectra with the buffer were subtracted from the sample spectra. The percentage of α-helical structure was estimated from the far UV CD spectrum according to the method of Luo and Baldwin [[3](#_ENREF_3)]. For binding experiments mgp41 was mixed with an equimolar amount of a synthetic gp41 peptide of sequence 110-141 (CP-110-141) [[1](#_ENREF_1)] and the near UV spectrum was recorded and compared to the isolated mgp41. The peptide was purchased to EZ Biolab (Carmel, IN, USA). The thermal stability of wt and mutant gp41 ectodomains was analyzed by differential scanning calorimetry using a Microcal VP-DSC microcalorimeter (Microcal Inc., Northampton, MA). Thermal scans were made at 90°C·h^−1^. Instrumental baselines were recorded with pure buffer and subtracted from the sample scans. Partial heat capacity data were calculated using Origin software (Originlab) and normalized per mole of protein monomer.

**References**

[1] He Y, Cheng J, Li J, Qi Z, Lu H, Dong M, et al. Identification of a critical motif for the human immunodeficiency virus type 1 (HIV-1) gp41 core structure: implications for designing novel anti-HIV fusion inhibitors. Journal of virology 2008 Jul;82(13):6349-58.

[2] Peisajovich SG, Blank L, Epand RF, Epand RM, Shai Y. On the interaction between gp41 and membranes: the immunodominant loop stabilizes gp41 helical hairpin conformation. Journal of molecular biology 2003 Mar 7;326(5):1489-501.

[3] Luo P, Baldwin RL. Mechanism of helix induction by trifluoroethanol: a framework for extrapolating the helix-forming properties of peptides from trifluoroethanol/water mixtures back to water. Biochemistry 1997 Jul 8;36(27):8413-21
